# Supplementary material for: Neural pathways and computations that achieve stable contrast processing tuned to natural scenes
Source: Nat Commun. 2024 Oct 3;15:8580. doi: 10.1038/s41467-024-52724-5 (PMC11450186; doi:10.1038/s41467-024-52724-5)
Supplement: Supplementary file 3 — Reporting Summary [file 41467_2024_52724_MOESM3_ESM.pdf]

nature portfolio

Corresponding author(s): Dr. Marion Silies

Last updated by author(s): 8/26/2024

Reporting Summary

Nature Portfolio wishes to improve the reproducibility of the work that we publish. This form provides structure for consistency and transparency in reporting. For further information on Nature Portfolio policies, see our Editorial Policies and the Editorial Policy Checklist.

Statistics

For all statistical analyses, confirm that the following items are present in the figure legend, table legend, main text, or Methods section.

n/a	Confirmed	
☐	☒	The exact sample size (*n*) for each experimental group/condition, given as a discrete number and unit of measurement
☐	☒	A statement on whether measurements were taken from distinct samples or whether the same sample was measured repeatedly
☐	☒	The statistical test(s) used AND whether they are one- or two-sided<br>*Only common tests should be described solely by name; describe more complex techniques in the Methods section.*
☐	☒	A description of all covariates tested
☐	☒	A description of any assumptions or corrections, such as tests of normality and adjustment for multiple comparisons
☐	☒	A full description of the statistical parameters including central tendency (e.g. means) or other basic estimates (e.g. regression coefficient) AND variation (e.g. standard deviation) or associated estimates of uncertainty (e.g. confidence intervals)
☐	☒	For null hypothesis testing, the test statistic (e.g. *F*, *t*, *r*) with confidence intervals, effect sizes, degrees of freedom and *P* value noted<br>*Give P values as exact values whenever suitable.*
☒	☐	For Bayesian analysis, information on the choice of priors and Markov chain Monte Carlo settings
☒	☐	For hierarchical and complex designs, identification of the appropriate level for tests and full reporting of outcomes
☒	☐	Estimates of effect sizes (e.g. Cohen's *d*, Pearson's *r*), indicating how they were calculated

Our web collection on statistics for biologists contains articles on many of the points above.

Software and code

Policy information about availability of computer code

Data collection	Bruker Prairie View for 2p imaging data acquisition, Flywire.ai for connectomics work.
Data analysis	Custom code in Matlab (for Dm12 2p imaging analysis) and Python 2.7 (for 2p imaging) and Python 3.9 (for connectomics analysis) was used for data analysis and plotting. The code is available on Github: https://github.com/silieslab/Gur-etal-2024<br>Python tools: Anaconda, Visual Studio Code<br>Python packages: Built-in modules, SIMA (Kaifosh et. al, 2014), numpy, pandas, matplotlib, scipy, sklearn, statsmodels, pickle, xml, fafbseg<br>Matlab tools: signal processing, image processing, statistics and machine learning toolboxes

For manuscripts utilizing custom algorithms or software that are central to the research but not yet described in published literature, software must be made available to editors and reviewers. We strongly encourage code deposition in a community repository (e.g. GitHub). See the Nature Portfolio guidelines for submitting code & software for further information.

Data

Policy information about availability of data

All manuscripts must include a data availability statement. This statement should provide the following information, where applicable:

- Accession codes, unique identifiers, or web links for publicly available datasets
- A description of any restrictions on data availability
- For clinical datasets or third party data, please ensure that the statement adheres to our policy

Source data of this study can be found on Zenodo: https://zenodo.org/doi/10.5281/zenodo.13327244 .

Research involving human participants, their data, or biological material

Policy information about studies with human participants or human data. See also policy information about sex, gender (identity/presentation), and sexual orientation and race, ethnicity and racism.

Reporting on sex and gender	This study did not involve human participants.
Reporting on race, ethnicity, or other socially relevant groupings	This study did not involve human participants.
Population characteristics	This study did not involve human participants.
Recruitment	This study did not involve human participants.
Ethics oversight	This study did not involve human participants.

Note that full information on the approval of the study protocol must also be provided in the manuscript.

Field-specific reporting

Please select the one below that is the best fit for your research. If you are not sure, read the appropriate sections before making your selection.

☒ Life sciences ☐ Behavioural & social sciences ☐ Ecological, evolutionary & environmental sciences

For a reference copy of the document with all sections, see nature.com/documents/nr-reporting-summary-flat.pdf

Life sciences study design

All studies must disclose on these points even when the disclosure is negative.

Sample size	Sample-sizes for imaging experiments were chosen based on typical sample sizes in the field (e.g. Serbe et. al (2016) Neuron, Strother et al. (2017) Neuron, Fisher et al. (2015) Curr. Bio.). All sample sizes are provided in the figure legends.<br>Connectomics data comes from 700 Tm9 neurons from a single Drosophila melanogaster.
Data exclusions	For 2p imaging data, recordings with strong motion artifacts were discarded. Regions of interests were further filtered using response amplitude and response reliability as mentioned in detail in the manuscript Methods section.<br>For connectomics data, connections less than 3 synaptic contacts were excluded.
Replication	2p imaging datasets for the characterization of luminance gain for L2, L3, Tm1 and Tm9 were replicated over several years. All attempts at replication were successful.
Randomization	For 2p experiments, flies of different genotypes were recorded within similar time frames using the same experimental protocol. Stimulus presentation trials were pseudo-randomized.
Blinding	The study was not blinded, the same experimental procedure applied to all groups within a dataset.

Reporting for specific materials, systems and methods

We require information from authors about some types of materials, experimental systems and methods used in many studies. Here, indicate whether each material, system or method listed is relevant to your study. If you are not sure if a list item applies to your research, read the appropriate section before selecting a response.

Materials & experimental systems

n/a	Involved in the study
☒	☐ Antibodies
☒	☐ Eukaryotic cell lines
☒	☐ Palaeontology and archaeology
☐	☒ Animals and other organisms
☒	☐ Clinical data
☒	☐ Dual use research of concern
☒	☐ Plants

Methods

n/a	Involved in the study
☒	☐ ChIP-seq
☒	☐ Flow cytometry
☒	☐ MRI-based neuroimaging

Animals and other research organisms

Policy information about studies involving animals; ARRIVE guidelines recommended for reporting animal research, and Sex and Gender in Research

Laboratory animals

Drosophila melanogaster from 1 to 8 days post eclosion were used in this study. Strains:
D. melanogaster: L3: MH56-Gal4
D. melanogaster: L2: 21Dhh-Gal4
D. melanogaster: L1: c202a-Gal4
D. melanogaster: Tm1: R74G01-Gal4
D. melanogaster: Tm2: R28D05-p65ADZp ; R82F12-ZpGdbd

D. melanogaster: Tm4: R53C02-p65ADZp ; R60H04-ZpGdbd
D. melanogaster: Tm9 LexA: 24C08-LexA
D. melanogaster: Tm9 Gal4: R24C08-Gal4
D. melanogaster: T4-T5: R59E08-LexA
D. melanogaster: Dm12: SS00359 split Gal4
D. melanogaster: UAS-GCaMP6f
D. melanogaster: lexAop-GCaMP6f
D. melanogaster: UAS-iGluSnFR
D. melanogaster: GluClαFlpSTOP-ND
D. melanogaster: UAS-Flp
D. melanogaster: GluClαMI14426
D. melanogaster: UAS-CsChrimson::mVenus
D. melanogaster: ort [1], ninaE[1]

Wild animals

This study did not involve wild animals.

Reporting on sex

The FAFB dataset is an EM reconstruction of one female fly. 2p experiments are also done using female flies except the optogenetics experiments where blind male flies were used due to ease of generating male flies with first chromosome mutations that lead to blindness. There is no known relevance of sex to the subject of investigation of this study. No known sexual dimorphism exists in the fly peripheral visual system.

Field-collected samples

This study did not involve field-collected samples.

Ethics oversight

No ethical approval is required for research on Drosophila melanogaster. The laboratory has all the permits to work with genetically modified organisms.

Note that full information on the approval of the study protocol must also be provided in the manuscript.

Plants

Seed stocks

This study did not involve seed stocks.

Novel plant genotypes

This study did not involve novel plant genotypes.

Authentication

This study did not involve plants.
